# COV-MobNets: a mobile networks ensemble model for diagnosis of COVID-19 based on chest X-ray images

**DOI:** 10.1186/s12880-023-01039-w

**Published:** 2023-06-15

**Authors:** Mohammad Amir Eshraghi, Ahmad Ayatollahi, Shahriar Baradaran Shokouhi

**Affiliations:** School of Electrical Engineering, Iran University of Science and Technology, Tehran, Iran

**Keywords:** MobileNetV3, MobileViT, COVID-19 diagnosis, Chest X-ray images, Ensemble learning, Deep learning

## Abstract

**Background:**

The medical profession is facing an excessive workload, which has led to the development of various Computer-Aided Diagnosis (CAD) systems as well as Mobile-Aid Diagnosis (MAD) systems. These technologies enhance the speed and accuracy of diagnoses, particularly in areas with limited resources or remote regions during the pandemic. The primary purpose of this research is to predict and diagnose COVID-19 infection from chest X-ray images by developing a mobile-friendly deep learning framework, which has the potential for deployment in portable devices such as mobile or tablet, especially in situations where the workload of radiology specialists may be high. Moreover, this could improve the accuracy and transparency of population screening to assist radiologists during the pandemic.

**Methods:**

In this study, the Mobile Networks ensemble model called COV-MobNets is proposed to classify positive COVID-19 X-ray images from negative ones and can have an assistant role in diagnosing COVID-19. The proposed model is an ensemble model, combining two lightweight and mobile-friendly models: MobileViT based on transformer structure and MobileNetV3 based on Convolutional Neural Network. Hence, COV-MobNets can extract the features of chest X-ray images in two different methods to achieve better and more accurate results. In addition, data augmentation techniques were applied to the dataset to avoid overfitting during the training process. The COVIDx-CXR-3 benchmark dataset was used for training and evaluation.

**Results:**

The classification accuracy of the improved MobileViT and MobileNetV3 models on the test set has reached 92.5% and 97%, respectively, while the accuracy of the proposed model (COV-MobNets) has reached 97.75%. The sensitivity and specificity of the proposed model have also reached 98.5% and 97%, respectively. Experimental comparison proves the result is more accurate and balanced than other methods.

**Conclusion:**

The proposed method can distinguish between positive and negative COVID-19 cases more accurately and quickly. The proposed method proves that utilizing two automatic feature extractors with different structures as an overall framework of COVID-19 diagnosis can lead to improved performance, enhanced accuracy, and better generalization to new or unseen data. As a result, the proposed framework in this study can be used as an effective method for computer-aided diagnosis and mobile-aided diagnosis of COVID-19. The code is available publicly for open access at https://github.com/MAmirEshraghi/COV-MobNets.

## Introduction

Severe Acute Respiratory Syndrome Coronavirus-2 (SARS-CoV-2) was initially identified in Wuhan, China, in December 2019, and then swiftly spread worldwide. Millions of individuals throughout the world have been impacted by COVID-19 [1–4]. Due to the high contagiousness of COVID-19, it is essential to quickly screen, identify, and isolate patients in order to stop the disease’s transmission and hasten their effective treatment [3, 4].

Despite the fact that the Reverse Transcription Polymerase Chain Reaction (RT-PCR) test plays a key role in diagnosing COVID-19, a high rate of disease progression and aggravation, particularly in Omicron and Delta variants, was caused by the lengthy turnaround time for RT-PCR results [5, 6]. However, until the PCR test result is identified, those who have the disease can spread it to a large number of people as a disease transmission source. In addition, studies on COVID-19 variants have shown that the RT-PCR test has a significant rate of false negatives, even for patients with severe symptoms [2, 4, 5, 7]. In these situations, COVID-19 should be identified by medical imaging methods, such as computed tomography (CT) and Chest X-ray (CXR) scans, which have been shown to diagnose infection accurately [3, 8, 9].

Although CT has been proven to be accurate diagnostic techniques for COVID-19 detection [10, 11], it has several significant drawbacks, such as the huge cost, the inability to be done as a bedside test, and about 70 times more ionizing radiation than X-ray [2, 1]. As per the recommendation provided by the American College of Radiology, CT scans should not be the primary diagnostic modality to be employed [12]. The CXR is a widely available, quick, cheap, non-invasive method, and a widespread clinical technique for the diagnosis of COVID-19 [8, 13]. However, accurate X-ray image diagnosis requires expertise and compared to other imaging techniques, the diagnosis of COVID-19 infection from a chest X-ray is far more challenging [1].

Researchers are investigating Artificial Intelligence (AI)-based methods for COVID-19 identification utilizing X-ray images because of the successful and effective use of Deep Learning (DL) techniques in the field of Computer Vision and the biomedical [3, 4], because of the capability of successfully modeling higher-order systems and achieving human-like performance [14]. DL techniques have been used in numerous studies to automate the diagnosis of COVID-19 [1, 2, 5], [15–17]. In the field of computer vision, the DL-based model usually takes advantage of a hierarchical structure of Convolutional Neural Network (CNN) or transformer encoder module in Vision Transformer (ViT) [18] structure. These central blocks can extract the features related to COVID-19 infection from X-ray images. Because of the automatic feature learning ability of DL methods, COVID-19 classification based on deep neural networks is being widely used [3]. Computer-Aided Diagnosis (CAD) systems utilize advanced machine learning algorithms and image processing techniques to analyze X-ray images and deliver timely and reliable results, thereby aiding in the early detection, monitoring, and management of COVID-19 cases [19].

To highlight recent developments in the diagnosis and detection of COVID-19 based on DL, several investigations are presented. Ghaderzadeh et al. [5] proposed a framework for COVID-19 detection based on two phases of CNN models. The first phase classifies COVID-19 X-ray images by using DenseNet201 as a feature extractor. The dataset contains 10,816 public images and 341 local images of two categories: normal and COVID-19 samples. X-ray and CT-scan images were classified by the proposed framework. In another research work, Duran-Lpez et al. [2] proposed COVID-XNet, comprising five convolutional layers, which was a custom DL system to detect and locate COVID-19 in chest X-ray images. The dataset includes 2,589 and 4,337 images were considered for COVID-19 and normal classes, respectively. This study took advantage of a histogram matching process for similarity in terms of histogram distribution, rib suppression, and a contrast enhancement method in other to reduce the large variability of X-ray images and enhance the relevant information.

Further, Afshar et al. [15] proposed a COVID-CAPS architecture consisting of 4 convolutional layers and 3 Capsule layers which classify positive and negative COVID-19. The dataset is generated from two public chest X-ray datasets. This research highlights that COVID-CAPS contains fewer trainable parameters compared to state-of-the-art models. In another research, the Convolutional Neural Network (CNN) is used by Wang et al. [16] to detect pneumonia, COVID-19, and normal cases from each other. The research proposed the COVID-Net network, which uses a lightweight residual projection-expansion design pattern. The dataset comprises 13,975 CXR images across 13,870 patient cases. This architecture’s parameter is only 11.75 million and the overall accuracy is 93.3%.

Afterward, Waheed et al. [17] suggested CovidGAN which takes advantage of a Generative Adversarial Network (GAN) to increase the CXR data by synthetic augmentation. The target of the study is to improve COVID-19 detection on the VGG network. The dataset is composed of 1,124 CXR images, which comprise COVID-CXR and Normal-CXR. This study indicates the importance of augmentation techniques based on GAN networks. Wang et al. [20] proposed MLES-Net which takes advantage of the correlation between global and local features to generate the attention mask, which can focus automatically on the important points in various information, leading to improvement of the model efficiency. This research combines two publicly CXR dataset, containing 760 and 5863 X-Ray images related to COVID-19, normal, and pneumonia.

Samee et al. [21] proposed a novel metaheuristic approach based on hybrid dipper-throated and particle swarm optimizers to optimize VGG19 deep network for the detection of COVID-19 from X-ray images. In this study, lung region segmentation and augmentation were applied to the dataset, and a feature selection method was used to select the most significant features that can boost the classification results. Further, Elzeki et al. [22] suggested CXRVN network based on convolution neural networks which used three different COVID-19 X-ray datasets. They adopt GANs to construct artificial instances for further data augmentation.

In this study, the researchers developed the Covid-19 classification model based on deep learning and ensemble learning, taking the advantage of CXR images. The positive COVID-19 in the input X-ray images is predicted by the diagnosis model. The author proposed a novel model called COV-MobNets, based on an ensemble of two lightweight and mobile-friendly deep networks for the classification of COVID-19 based on X-ray images with improved accuracy. Image augmentation methods are applied to address the issue of over-fitting during the training process.

The background and motivation for developing Computer-Aided Diagnosis (CAD) and Mobile-Aid Diagnosis (MAD) systems for the detection of COVID-19 from CXR images stems from the necessary need for accurate and efficient diagnostic tools during the global pandemic. These systems have the potential to be implemented not only on computers, but also on mobile phones with limited hardware capacity. The primary purpose of the proposed COV-MobNets model in the context of COVID-19 diagnosis is to provide an efficient and accurate tool for automated detection and classification of COVID-19 cases from CXR images, especially in situations where the workload of radiology specialists may be high. With the aid of deep learning algorithms and mobile technology, COV-MobNets aims to enhance the accuracy of COVID-19 diagnosis which can be suitable for deployment on mobile devices.

This paper has been formed into 4 sections. The proposed method, preprocessing techniques, and datasets employed for experiments are described in section 2, and Section 3 presents and discusses experimentation results. Finally, the conclusion is presented in Section 4.

## Materials and methods

### Dataset

The dataset used for this research is COVIDx-CXR-3 [23] benchmark dataset, which is a large-scale benchmark dataset of CXR images for supporting COVID-19 computer vision research, contains 30.386 CXR images from 17.036 patients from at least 51 countries. COVIDx-CXR-3 provides a smaller and well-balanced test set achieved by sampling a random 8:2 patient split from the RSNA RICORD initiative to ensure networks are evaluated against expertly annotated positive samples [23].

This dataset is not only one of the newest and largest, but it also creates a relatively balanced train and test sets for SARS-COV-2 positive and negative detection in terms of image count. Table 1 demonstrates the details of COVIDx-CXR-3 benchmark dataset. This research randomly split the train set into a 9:1 ratio for the training and validation sets, respectively, and evaluated the proposed model using the unique expertly annotated test set provided by dataset.

**Table 1:** Data distribution used for training and evaluating the proposed model

Type	classes	No. of samples	No. of patients
**Train**	Positive Negative	15.994	2.808
		13.992	13.850
**Test**	Positive Negative	200	178
		200	200

### Data preparation and normalization

Research has shown that not only deep learning algorithms but also image processing techniques can play a prominent role in the extraction of hidden patterns, particularly in medical image data [5]. In the first stage, the dataset images are decoded and converted to the common RGB format by repeating x-ray grayscale images three times. Then, the images are resized to (128,128) pixels. It is suitable to normalize images before the model can be adequately trained on them. Commonly, an RGB image has three channels that have a pixel intensity value, ranging from dark to white (0 to 255). Proposed three channel Images are normalized such that they have a value between 0.0 and 1.0 before feeding to the model’s input.

### Online data augmentation

In research that uses deep learning algorithms, they depend on numerous data to escape from the important challenge of model over-fitting [24]. Over-fitting is a condition when the model learns from the training data effectively, but it is unable to function well in untrained data. In this situation, the model begins to learn some complicated patterns in each example that are not necessarily generalizable to the others [25]. Therefore, at this stage, data augmentation was applied to the training dataset, using transformations such as Vertical flip and Horizontal flip. The process of data augmentation was implemented in an online manner on the COVIDx-CXR-3 dataset, wherein it was randomly applied to each batch of the training set during the training phase. This technique enables the model to generalize agreeably and be more robust to new cases.

### Proposed method

Our study takes the advantage of lightweight networks to guarantee minimal parameter demand and real-time detection performance. This section presents the MobileViT model and MobileNetV3 models used in the proposed Mobile Networks ensemble model.

#### MobileNetV3 

Convolutional Neural Network (CNN) architectures have been suggested to address a variety of computer vision issues, and can enhance performance of the models in terms of accuracy, size, efficiency, and speed [26]. The main layers of CNN in the classification mode are a combination of convolutional layers, pooling layers, and activation functions which can act as a feature extractor, and fully connected layers that can act as a feature classifier [27]. The pooling layer is considered as a down-sampling technique used to preserve crucial information about image information, and it also decreases the extracted features’ dimensionality [25]. The central structure of CNN is convolutional layers, demonstrated in Eq. 1.

1 $$y\left(t\right)= {\int }_{-\infty }^{\infty }x\left(p\right)h\left(t-p\right)dp=x\left(t\right)*h\left(t\right)$$

Howard et al. [28] proposed MobileNetV3, which was improved from MobileNetV1 [29] and MobileNetV2 [30]. They used a network architecture search (NAS) technique called NetAdapt algorithm that was used to search for the best kernel size and find the optimized MobileNet architecture to comply with platforms that have low-resourced hardware. Moreover, a novel nonlinearity known as hard swish (h-swish) is included in MobileNetV3. Equations 2 and 3 defines the h-swish nonlinearity, which is used to minimize the number of training parameters and decrease the size and complexity of the model.

2 $$h-swish\left(x\right)= x . \sigma \left(x\right)$$

3 $$\sigma \left(x\right)= \frac{ReLU6(x+3)}{6}$$

**Fig. 1:**
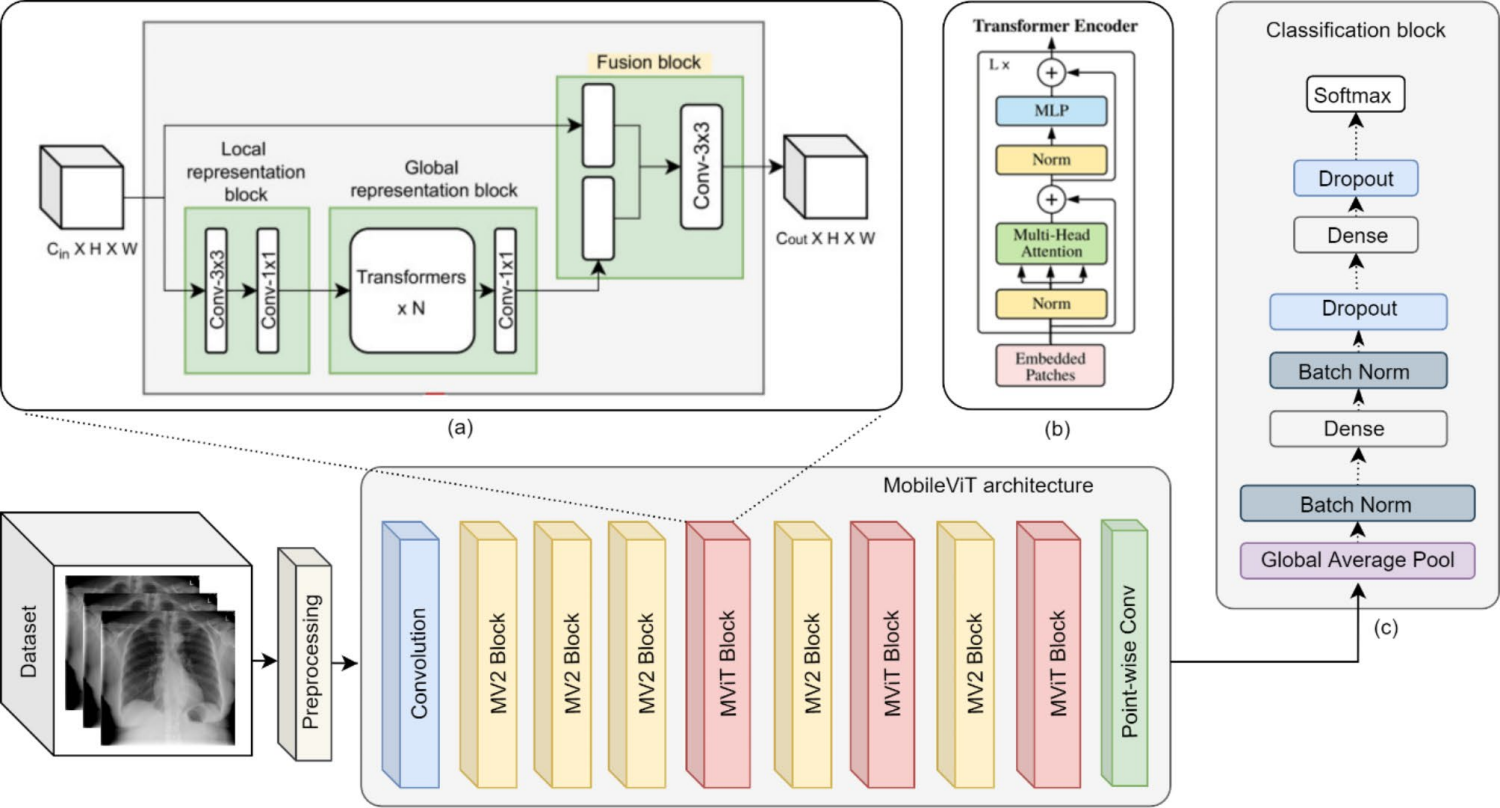
The overall overview of the improved MobileNetV3 architecture, comprising **(a)** MobileNetV3 block; **(b)** Depthwise separable convolution block; **(c)** Squeeze-and-excitation block; **(d)** Classification block

The structure of a MobileNetV3 block, presented in Fig. 1a, contains inverted residual blocks as a main unit, which uses a skip connection for connecting the feature of input and output on the identical channels, resulting in enhanced features representation with less memory consumption. The inverted residual block consists of two important blocks: a squeeze-and-excitation (SE) block and a depthwise separable convolution block. A significant method in multiple computer vision applications is an efficient CNN that implements the depthwise convolution structure and is known for its quick training process [26]. The depthwise convolutional kernel is a learnable parameter and plays a key role in extracting spatial features. Moreover, it improves model effectiveness and lower computing costs [28]. The depthwise separable convolution block (Fig. 1b) consists of a kernel of 3 × 3 depthwise convolution used for input channels separately and a 1 × 1 pointwise convolution kernel. The batch normalization layer (BN) and the h-swish activation function are used after these kernels. During training, SE block is utilized to pay more attention to the pertinent features on each channel, resulting in improving feature representation [28, 26].

In the present study, the MobileNetV3-Large model, which is a light-weight, and mobile-friendly CNN-based network, was implemented for extracting features of COVID-19 CXR images. The classification task is done by adding layers of global average pooling, fully connected, batch normalization, dropout, and softmax function. The overall framework of improved MobileNetV3 is demonstrated in Fig. 1.

#### MobileViT 

MobileViT, proposed by Sachin et al. of Apple in 2021 [31], is a general-purpose, lightweight, and mobile-friendly vision transformer (ViT). MobileViT incorporates the architectures of ViTs and CNNs. Hence, it not only has the self-attention and global vision of transformer networks, but it also has the efficiency and lightweight of CNNs, which allows to learn global and local features strongly. The MV2 block and MVIT block are the central units of the MobileViT architecture.

In MobileNetV2 [30], the linear bottleneck inverse residual block known as MV2 was proposed. With this structure, low-dimensional compressed data is expanded to higher dimensions, depthwise separable convolution is used to filter the data, and the linear bottleneck block is used to return the features to the lower dimensions [32]. The process in this structure makes use of tiny tensor data, which decreases the demand on the embedded hardware for main memory access and increases response time.

The structure of MViT block, indicated in Fig. 3a, can be divided into 3 modules: the local information coding module, the global information coding module, and the feature fusion module, which act as a local feature extractor, global feature extractor, and fuse feature information, respectively. MVIT can entirely extract the image features with fewer parameters [31, 33].

**Fig. 2:**
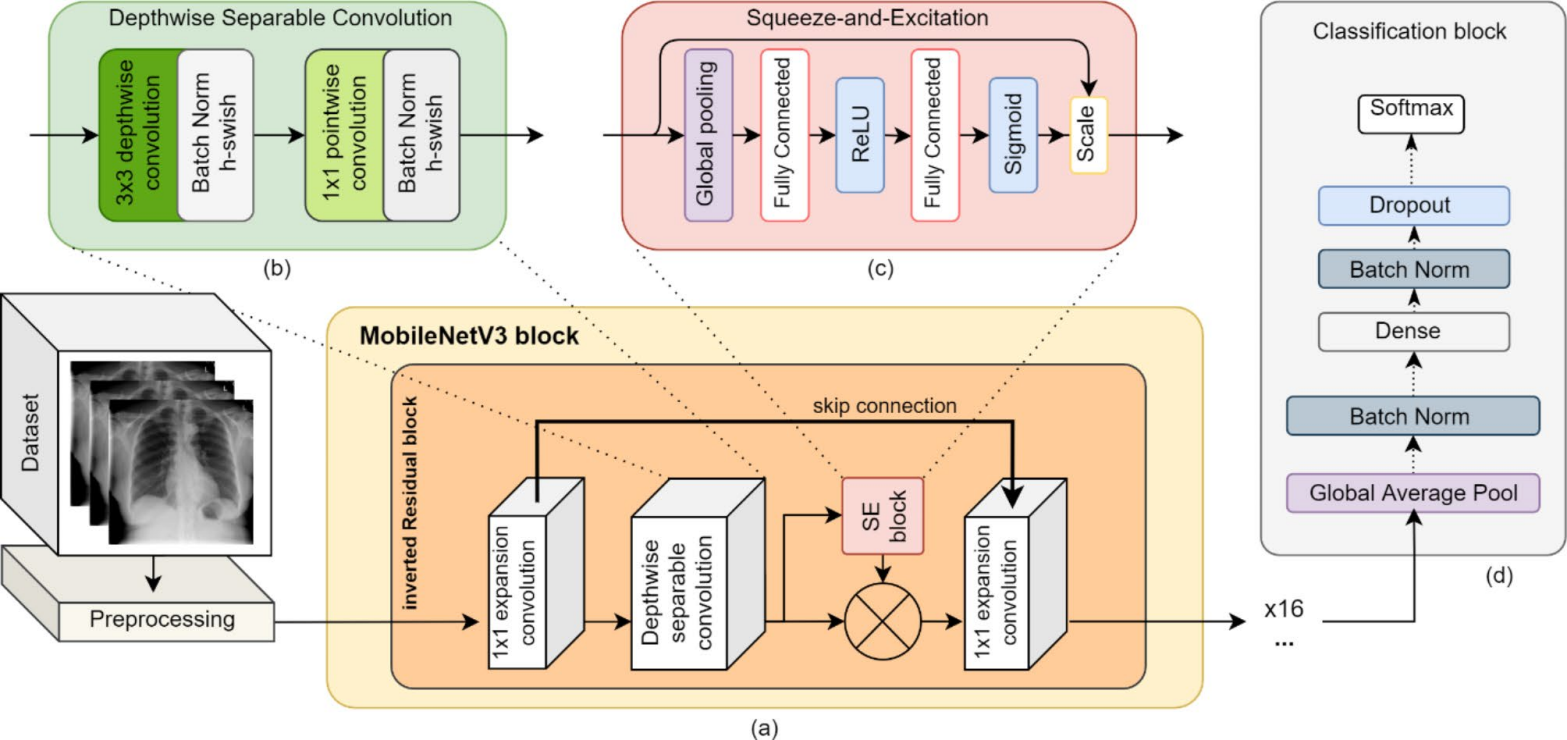
Overall overview of proposed MobileViT architecture consist of: **(a)** MobileViT block; **(b)** Transformer Encoder block; **(c)** Classification block

MobileViT uses a transformer structure in a specific way. Natural Language Processing (NLP) frequently makes use of transformer structures that comprise encoders and decoders components, but transformer structures in ViTs consist of only an encoder component [18]. The encoder components, demonstrated in Fig. 2b, are collected from a stack of similar encoders and each one includes a multihead attention layer, normalization layers, feed forward layer, and residual connection structure [18]. Transformer architectures have incorporated self-attention mechanisms to effectively capture the long-range dependencies between input images, leading to significant improvements in performance [34]. A type of self-attention structure known as Multihead Attention that enables the model to focus on various aspects of information [35]. The multihead attention formulas are shown in formulas 4–6. The terms Q, K, V, and W respectively stands for the query vector, key vector, value vector, and weight matrix [35].

4 $$\begin{array}{l}{Q_i} = QW_i^Q,\\{K_i} = KW_i^K,\\{V_i} = VW_i^V,\\\,\,\,\,\,\,\,\,\,\,\,\,\,\,\,\,\,\,\,\,\,{\rm{i}} = 0, \ldots ,8.\end{array}$$

5 $${head}_{i}= Attention\left({Q}_{i},{K}_{i},{V}_{i}\right), \text{i}=0, \dots ,8$$

6 $$\begin{array}{l}MultiHead\left( {Q,K,V} \right) = \\Concact\left( {hea{d_1}, \ldots ,hea{d_8}} \right){W^0}\end{array}$$

MobileViT applies an n*n standard convolution plus a point-wise convolution to an input tensor of the shape H*W*C. The outcome of this is a tensor of the form H*W*d. The tensor is then divided into h*w*d nonoverlapping patches. Then, each patch unfolded, resulting in intermediate-level embeddings of shape P*N*d, where P = w*h and N = H*W/P. Finally, the transformer is applied to these embeddings [31]. This process is illustrated in Fig. 2a.

In this paper, the MobileViT-XS model is implemented for extracting features of COVID-19 chest X-ray images, which is a light-weight, and mobile-friendly transformer-based network. The classification task is done by adding layers of global average pooling, fully connected, batch normalization, dropout, and softmax function. The overall framework of improved MobileViT is demonstrated in Fig. 2.

#### Mobile Networks Ensemble Model

The ensemble model is a technique to enhance performance and raise the accuracy rate of the model. If there are more distinct differences between the models in terms of feature extraction method, the model’s performance will be better after the ensemble [25]. By utilizing multiple feature extractors, the model is able to learn a more robust representation of the data, which allows it to better generalize to new or unseen data. This is particularly important in the context of COVID-19 image classification, where the availability of data is limited and the need for accurate and reliable classification is paramount. Hence, the features of chest X-ray images are extracted using two separate approaches, which can widely represent the differences between COVID-19 X-ray images widely and can improve classification outcomes.

The proposed framework combines two different feature extraction models, which are considered light-weight and mobile-friendly models. The MobileViT model base on transformer structure and MobileNetV3 model based on convolutional networks extract the features of chest X-ray images and classify them into positive and negative COVID-19 categories.

**Fig. 3:**
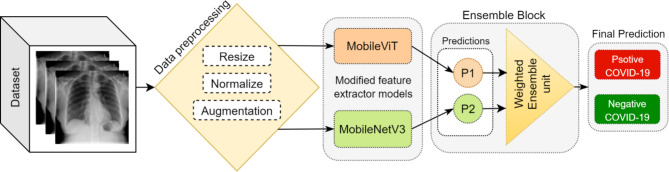
Overall framework of COV-MobNets (proposed model)

**Fig. 4:**
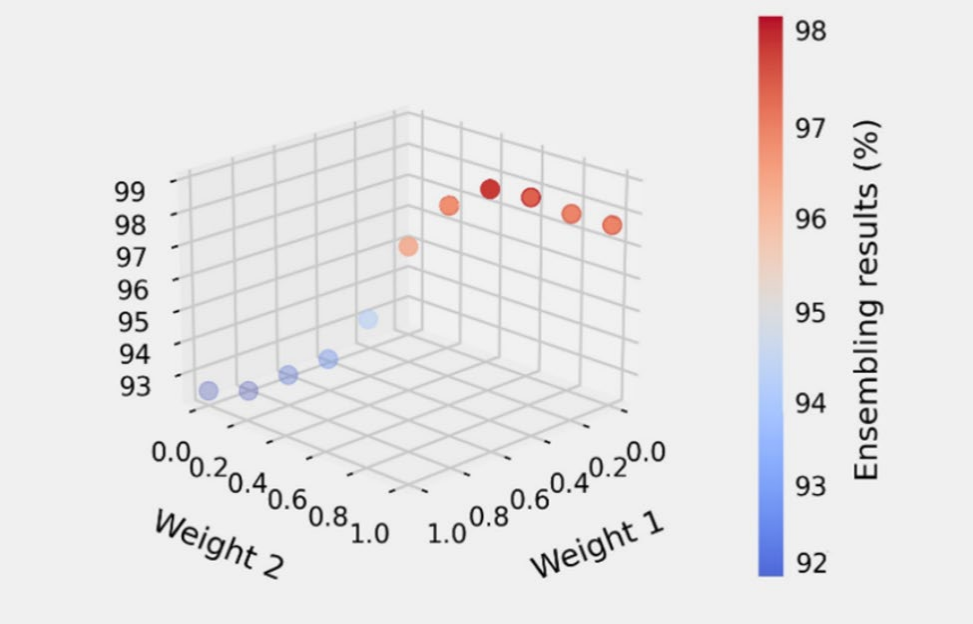
Ensemble results for different weight combinations

The weighted sum technique is the ensemble approach utilized in this research. Different weight combinations are applied to find the best performance, as indicated in Fig. 4. Hence, the MobileNetV3 model’s output results are multiplied by a coefficient of 0.7, and the MobileViT model’s output results are multiplied by a coefficient of 0.3, which are considered as weight 2 and weight 1, respectively. Finally, the sum of the two results is the final prediction outcome. Equation 7 shows the final prediction equation of the ensemble model. Figure 3 shows the overall framework of the proposed Mobile Networks ensemble model called COV-MobNets for COVID-19 CXR image classification.

7 $$Final Prediction= W1. P1\left(x\right)+W2.P2\left(x\right)$$

## Result and discussion

### Metrics

Metrics The sensitivity, specificity, accuracy, and F1 score assessment criteria were established based on the model’s performance using a confusion matrix in order to objectively assess the performance of the proposed model. Sensitivity in this context was defined as the proportion of COVID-19 instances, which the model properly identified, to all of the actual COVID-19 cases. Specificity was defined as the proportion of the non–COVID-19 instances properly detected by the model to all the actual non–COVID-19 cases. Moreover, accuracy was determined as the percentage of all COVID-19 and non-COVID-19 instances that were correctly identified from the chest X-ray images. These evaluations were performed using the following equations 8–11.

8 $$\begin{array}{l}Sensitivity\left( {Recall} \right) = \\\,\,\,\,\,\,\,\,\,\frac{{TP}}{{True\,Positive + False\,Negative}}\end{array}$$

9 $$Specificity= \frac{True Negative}{True Negative+False Posetive}$$

10 $$Accuracy=\frac{True Negative+True Posetive }{Totall Cases}$$

11 $$Precision= \frac{True Positive}{True Positive+False Posetive}$$

### Experimental setup

The process of implementing the proposed method is as follows:

Step 1: The original images are resized to shape (128,128,3). Then, the dataset is divided into training and validation sets at a ratio of 9:1. Also, 400 balanced samples are defined separately in the dataset for the test set.

Step 2: the batch size is set to 32, and the Adam algorithm is used to optimize the models with an initial learning rate of 1 × 10 − 4.

Step 3: Data augmentation is employed on each batch of the training set during the training process. Both models are trained for 30 epochs, and the parameters of the trained models are saved.

Step 4: Two models with saved parameters are integrated into Mobile Networks ensemble model for testing.

Table 2 shows the parameter details of the models.

**Table 2:** Parameters of both models used in COV-MobNets for training process

Model	MobileViT	MobileNetV3
**Optimizer**	Adam	Adam
**Learning rate**	Exponentially decay	Exponentially decay
**Initial learning rate**	${10}^{-4}$	${10}^{-4}$
**Final learning rate**	${10}^{-6}$	${10}^{-5}$
**Loss**	Binary Cross Entropy	Binary Cross Entropy
**Epoch**	30	30
**Batch size**	32	32
**Image size**	128 × 128	128 × 128

### Experimental comparison

**Fig. 5:**
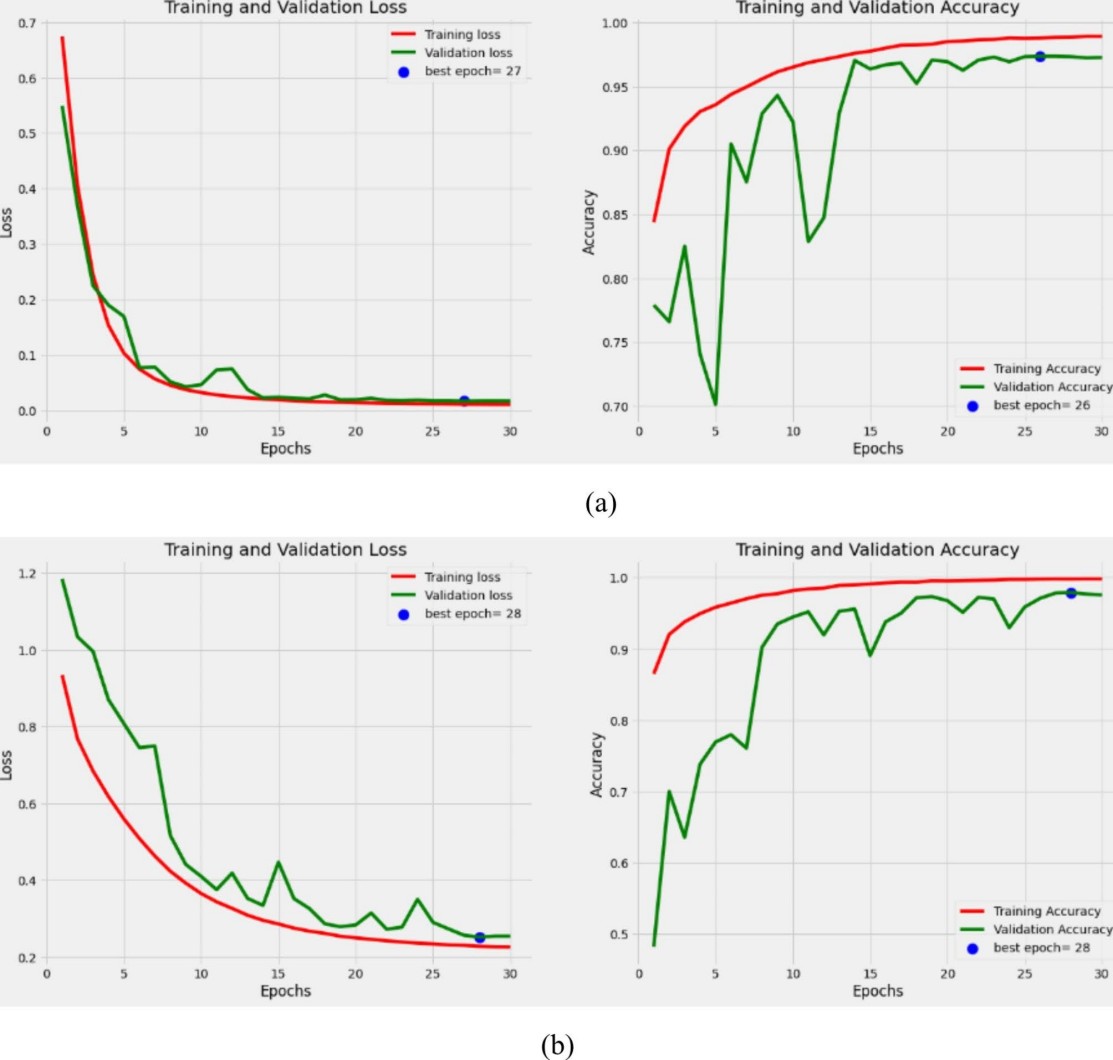
Loss and accuracy change curves of training and validation sets for: **(a)** the MobileViT model; **(b)** the MobileNetV3 model

The process outlined in the previous section was followed to conduct the experiment. The loss changes curve and the accuracy changes curve of the training set for the MobileViT and the MobileNetV3 model are shown in Fig. 5. Furthermore, Table 3 indicates rates of the accuracy, Sensitivity, Specificity, and precision of the MobileViT model, the MobileNetV3 model, and the Mobile ensemble model on the test set.

**Table 3:** Performance comparison for Covid-19 detection

Model	Data type	Number of classes	Image size	Accuracy (%)	Sensitivity (Recall) (%)	Specificity (%)	Precision (%)	F1 score
COVID-XNet [2]	X-ray	2	128 × 128	94.43	92.53	96.33	93.76	93.14
COVIDGAN [17]	X-ray	2	112 × 112	95	90	97.00	96%	---
COVID-CAPS [15]	X-ray	2	224 × 224	95.7	90	95.80	---	---
ResNet50 [1]	X-ray	2	224 × 224	96.1	91.8	96.6	76.5	83.5
MobileViT	X-ray	2	128 × 128	92.5	97.00	88.00	89.00	92.8
MobileNetV3	X-ray	2	128 × 128	97	97.50	96.50	96.53	97
COV-MobNets (Proposed)	X-ray	**2**	128 × 128	**97.75**	**98.50**	**97.00**	**97. 04**	**97.78**

It can be seen that the accuracy and precision of the MobileNetV3 based on CNN is 4.5% and 4.15% higher than that of the MobileViT model based on CNN and the Vision Transformer approach, respectively. Hence, the performance of the MobileNetV3 on the small dataset in terms of COVID-19 classification is better than second one. After being ensemble into the Mobile Networks ensemble model, rates of the accuracy and precision achieved 97.75% and 97.87%, respectively, showing enhanced performance.

**Fig. 6:**
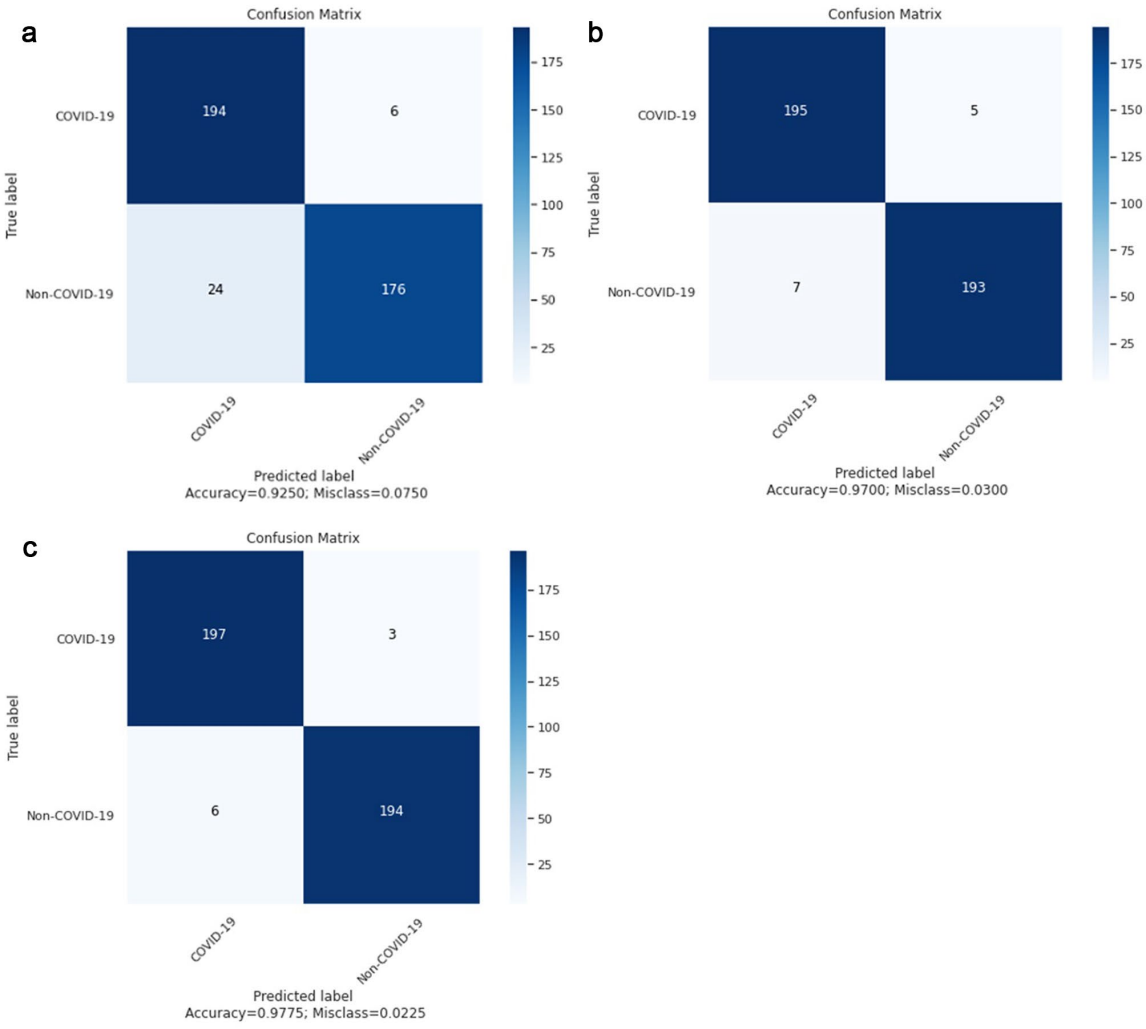
Confusion matrix of three models: **(a)** MobileViT model; **(b)** MobileNetV3 model; **(c)** COV-MobNets model (proposed)

The classification capabilities of the model may be evaluated by accuracy rate, but precise details cannot be displayed with that. The confusion matrix, which compares the predicted outcome and actual value and is called the comparison matrix, can clearly show the prediction information of each class when the trained model produces predictions. The classification performance of the ensemble model suggested in this paper is further examined using the confusion matrix. the confusion matrix of the three models is shown in Fig. 6.

The confusion matrixes indicate that the recognition capability of the MobileNetV3 model is more balanced than MobileViT, and its recognition accuracy of Covid-19 and non-COVID-19 cases is about the same. The MobileViT model has a difference in the ability to recognize COVID-19 cases and non- COVID-19 cases from X-ray images, whereas the ability to recognize COVID-19 cases remains strong. While recognizing COVID-19 cases in both base models is appropriate, a remarkable improvement is seen after integrating these two powerful mobile networks based on different feature extractors. The Mobile Networks ensemble model (COV-MobNets) not only displays a more robust ability to recognize COVID-19 and non- COVID-19 cases but also maintained its balance.

In practical applications, better analyses of CXR images can more accurately diagnose COVID-19, so Mobile Networks ensemble model (COV-MobNets) can in a prominent way assist in the diagnosis of COVID-19 from the X-ray images. The accuracy rate of the proposed model is 3.32%, 2.25%, 2.05%, and 1.65% higher than the accuracy rate of the COVID-XNET, COVID-GAN, COVID-CAPS, and ResNet50 models, respectively. Consequently, the Mobile Networks ensemble model (COV-MobNets) achieved better classification performance.

## Conclusion

In this study, a diagnostic method was proposed for the COVID-19 CXR image classification that could identify COVID-19 and non-COVID-19 cases using a Mobile Networks ensemble model to help medical professionals make a better diagnosis in the real world. The research employed COVIDx-CXR-3 dataset, and to address the issue of overfitting, the online data augmentation techniques were applied during the training phase. An overall framework is designed that incorporates the MobileViT (based on ViT) and MobileNetV3 (based on CNN) into the Mobile Networks ensemble model called COV-MobNets. The accuracy of this model in the classification of positive and negative COVID-19 cases reached 97.75%. The findings indicate that the COV-MobNets proposed in this research were more accurate and performed better than earlier models and had a balanced capacity for classification.

COV-MobNets can be integrated into existing medical imaging systems to assist radiologists in detecting COVID-19 from chest X-rays as the Computer-Aid Diagnosis. The model can quickly analyze large volumes of X-ray images, reducing the time and effort required for manual diagnosis. Moreover, COV-MobNets can have the potential to implement on Mobile phones and tablets by taking advantage of TensorFlow Lite (TF-Lite). The TF-Lite format is designed to execute models efficiently on devices, which reduces the file size and introduces optimizations that do not affect accuracy [36]. Hence, by implementing it on a mobile phone, it can be a dedicated radiology assistant in the diagnostic process.

The proposed COV-MobNets model presents certain limitations that need to be addressed in future research to improve its applicability and accuracy. One of these limitations is the potential data limitations that can affect the performance of the model. Collecting more diverse and representative datasets can improve the model’s performance and reduce its limitations. Another limitation is the need for real-world validation to ensure the accuracy and effectiveness of the model in practical clinical settings. Real-world validation can provide additional evidence for the model’s effectiveness and limitations. Additionally, implementing the model on edge devices can provide a significant advantage in terms of portability and accessibility. Therefore, future research can focus on developing lightweight models that will be implemented on edge devices to facilitate their use in remote and resource-limited settings.

The findings of this study have significant implications for the medical profession, particularly in the context of the ongoing COVID-19 pandemic and the excessive workload that healthcare professionals were facing. The use of lightweight and mobile-friendly models makes the system easily deployable in resource-limited settings and allows for faster diagnosis and treatment. Moreover, the implementation of this model could help alleviate the workload burden on healthcare professionals by providing a reliable and efficient tool for COVID-19 diagnosis. In conclusion, the findings of this study offer promising solutions to aid medical professionals in the ongoing fight against the COVID-19 pandemic and the challenges it poses to the healthcare industry.

## Data Availability

The used datasets were obtained from publicly open-source datasets from: https://www.kaggle.com/datasets/andyczhao/covidx-cxr2.
